# Do Inequalities in Neighborhood Walkability Drive Disparities in Older Adults’ Outdoor Walking?

**DOI:** 10.3390/ijerph14070740

**Published:** 2017-07-07

**Authors:** Razieh Zandieh, Johannes Flacke, Javier Martinez, Phil Jones, Martin van Maarseveen

**Affiliations:** 1 Faculty of Geo-Information Science and Earth Observation (ITC), University of Twente, P.O. Box 217, 7500 AE Enschede, The Netherlands; j.flacke@utwente.nl (J.F.); j.martinez@utwente.nl (J.M.); m.f.a.m.vanmaarseveen@utwente.nl (M.v.M.); 2 School of Geography, Earth and Environmental Sciences, University of Birmingham, Edgbaston, Birmingham B15 2TT, UK; p.i.jones@bham.ac.uk

**Keywords:** physical activity, GIS, GPS, facilities, qualitative, quantitative, perception, walking interview, multilevel/hierarchical analyses, healthy urban planning

## Abstract

Older residents of high-deprivation areas walk less than those of low-deprivation areas. Previous research has shown that neighborhood built environment may support and encourage outdoor walking. The extent to which the built environment supports and encourages walking is called “walkability”. This study examines inequalities in neighborhood walkability in high- versus low-deprivation areas and their possible influences on disparities in older adults’ outdoor walking levels. For this purpose, it focuses on specific neighborhood built environment attributes (residential density, land-use mix and intensity, street connectivity, and retail density) relevant to neighborhood walkability. It applied a mixed-method approach, included 173 participants (≥65 years), and used a Geographic Information System (GIS) and walking interviews (with a sub-sample) to objectively and subjectively measure neighborhood built environment attributes. Outdoor walking levels were measured by using the Geographic Positioning System (GPS) technology. Data on personal characteristics was collected by completing a questionnaire. The results show that inequalities in certain land-use intensity (i.e., green spaces, recreation centers, schools and industries) in high- versus low-deprivation areas may influence disparities in older adults’ outdoor walking levels. Modifying neighborhood land use intensity may help to encourage outdoor walking in high-deprivation areas.

## 1. Introduction

Outdoor walking refers to total walking for different purposes—including transport, recreation and exercise—in outdoor space. It is a type of physical activity and has certain benefits for healthy aging [1,2]. Therefore, physical activity guidelines recommend older adults to take outdoor walks [3,4]. Despite this widespread knowledge, there is prevalence of physical inactivity among majority of older adults [3,5], particularly among older residents of high-deprivation areas (areas with high levels of social and economic disadvantages) of cities [6,7]. It has been shown that older residents of high-deprivation areas walk less than those of low-deprivation areas [6,8]. These findings highlight the importance of promoting outdoor walking levels among older adults, particularly among older residents of high-deprivation areas.

To promote outdoor walking levels, a growing body of literature has addressed the link between the built environment and walking [9,10,11,12]. Although the influences of the built environment on walking are not yet well understood [13,14], transportation and urban planning research has identified some relationships between neighborhood built environment and outdoor walking [15,16,17,18]. It has been argued that neighborhood built environment may support and encourage residents, especially older adults [19], to walk. The extent to which the built environment supports and encourages walking is called walkability and it reflects a quality of the neighborhood [12,16].

Different neighborhood built environment attributes (e.g., residential density, street connectivity, traffic condition, and aesthetics) may influence neighborhood walkability [17]. Three neighborhood built environment attributes have been identified as key elements of neighborhood walkability [20,21,22,23]: residential density, land use (mix and intensity), and street connectivity (defined in Table 1). These three built environment attributes shape the overall design and structure of a neighborhood and are known as “macro built environment attributes” [24,25]. They have a synergy in creating a walkable neighborhood [26]. Some research on walkability has addressed “neighborhood retail density” (defined in Table 1), in addition to the attributes mentioned above [16,27].

High neighborhood residential density, land-use mix, (certain) land-use intensity, and retail density provide diverse attractive destinations—or places (e.g., green space)—for walking at close distances (Table 1). High neighborhood street connectivity offers short and diverse walking routes to these destinations/places (Table 1). Presence, proximity and attractiveness of destinations/places may give people reasons to go out and support them to walk for transport, recreation and exercise [16,28]. Proximity to destinations/places is especially important for older adults’ daily activities and may encourage these people to get walking into their routine [29,30].

Accordingly, findings on lower outdoor walking levels among older residents of high- versus low-deprivation areas trigger questions about neighborhood walkability: do older residents of high-deprivation areas have a less supportive neighborhood for outdoor walking than those of low-deprivation areas? How do neighborhood residential density, land-use mix and intensity, street connectivity, and retail density influence outdoor walking among older adults living in low- and high-deprivation areas? These questions are important for healthy urban planning aiming at creating walkable built environment for everyone [35]. Urban planners incorporate these questions under the context of spatial inequality: the uneven provision of urban opportunities and resources among urban areas with different levels of socioeconomic deprivation [36,37]. Identifying inequalities in neighborhood walkability and finding shortcomings for supporting older adults’ outdoor walking in high-deprivation areas may benefit urban planning interventions.

To date, much research on neighborhood walkability has focused on associations between neighborhood residential density, land-use mix and intensity, street connectivity, and retail density and older adults’ walking levels, but they have reported inconsistent results [14,29]. Inequalities in neighborhood walkability and their influences on older adults’ outdoor walking levels in low- and high-deprivation areas have been rarely studied. Moreover, the scant existing studies on older adults’ walking in low- and high-deprivation areas have focused on perceived neighborhood built environment attributes [8,37,38]. Using the perceived built environment has advantage of involving personal assessments of neighborhood built environment, but it may not reflect the actual built environment [2]. Objectively measured neighborhood built environment may better reflect actual neighborhood built environment conditions. However, objective measurement approaches have been used only in a few studies on older adults’ walking [39,40], and have been scarcely employed in studies on older adults’ walking in low- and high-deprivation areas.

Therefore, this study aims to examine inequalities in neighborhood walkability (i.e., residential density, land-use mix and intensity, street connectivity, and retail density) in high- versus low-deprivation areas and their possible influences on disparities in older adults’ outdoor walking levels. For this end, it involves both objectively measured and perceived neighborhood built environment attributes and answers two research questions:

(1) How (un)equal are neighborhood residential density, land-use mix and intensity, street connectivity, and retail density in high- versus low-deprivation areas?
(2) What are the relationships between neighborhood residential density, land-use mix and intensity, street connectivity, and retail density, and older adults’ outdoor walking levels?

## 2. Materials and Methods

The study was administered in Birmingham, a superdiverse city [41] of over one million residents in the United Kingdom, from 7 July to end of October 2012. A concurrent mixed-method design [42] was employed in this study. By applying this research design, the authors tried to enrich the quantitative examinations (on “how objectively measured neighborhood built environment attributes and outdoor walking levels are”) with qualitative evidence (on “how perceived neighborhood built environment attributes may, in the view of older adults, influence outdoor walking levels”). Accordingly, qualitative findings were employed to help to support and interpret the quantitative findings.

This study used the sample (*n* = 173) and data on outdoor walking levels from a previous research [37]. The Geographic Positioning System (GPS) technology was used to objectively measure outdoor walking levels and a questionnaire was used to collect data on personal characteristics.

We collected data on neighborhood built environment attributes by using a Geographic Information System (GIS) and walking interviews. Detailed information on collecting data on neighborhood built environment attributes is presented later in this study.

### 2.1. Selection of Low- and High-Deprivation Areas 

**Figure 1:**
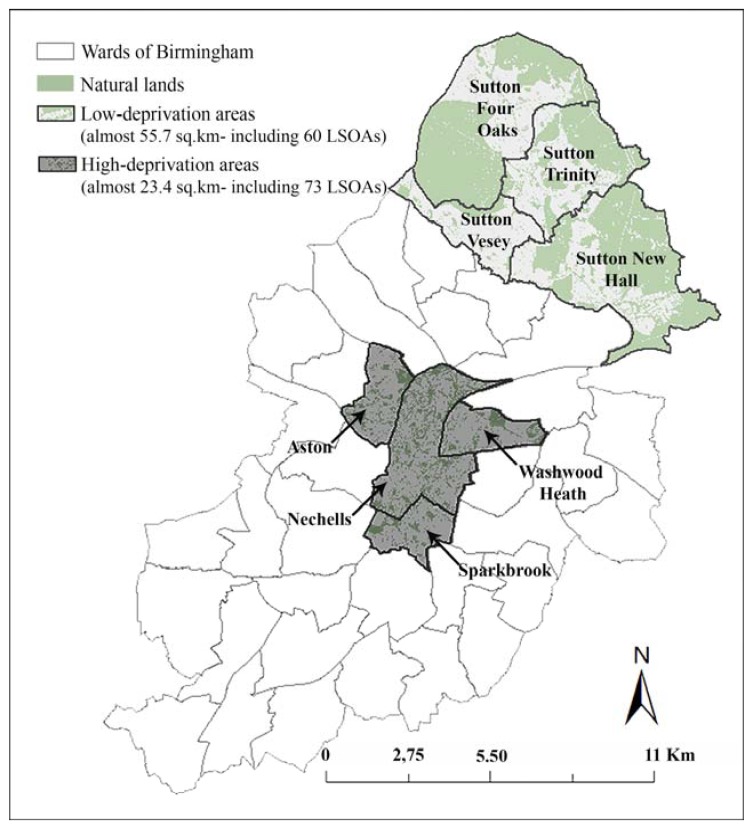
Locations of low- and high-deprivation areas in Birmingham. Adapted from [37].

The Index of Multiple Deprivation (IMD) was employed for identifying low- and high-deprivation areas on electoral ward scale [37]. The IMD is an aggregated score of seven domains of deprivation (i.e., income, employment, health and disability, education and skills, barriers to housing and services, crime, and living environment) used in the UK [6,43]. It is produced at the level of Lower Layer Super Output Areas (LSOAs): relatively homogenous geographic areas with 1500 residents on average [43,44]. Deprivation level for each ward was determined based on the ward’s area covered by the 20% most or 20% least deprived LSOAs. As a result, four low-deprivation areas and four high-deprivation areas were identified in Birmingham (Figure 1). Participants were recruited from these selected areas [37].

### 2.2. Participant Recruitment

A convenience sampling approach was applied to recruit participants from social centers (e.g., community centers, University of the Third Age, libraries, etc.) in all 8 selected wards [37]. Applying a convenience sampling approach is often the norm in health behavior studies on older adults [45]. By posting advertisements and arranging information sessions in social centers, older adults were informed about the research and process of participation in the research [37]. Inclusion criteria were being age 65 or over, resident of one of the selected wards, able to walk, independent in daily life activities, and mentally healthy. English speaking was not an eligibility criterion. A translator/assistant assisted participants (*n* = 58) who were non-English speakers or required help in filling the questionnaire. Quota sampling and UK census data (2001) were used to achieve maximum similarity to ethnic diversity in the total population of the selected wards. In total 216 participants received GPS tracking units, but 43 participants were excluded due to not using tracking units. Therefore, the final sample included 173 participants (*n* = 93 and *n* = 80 from low- and high-deprivation areas, respectively).

Based on participants’ availability and willingness to participate in walking interviews, a sub-sample was drown from the main sample [37]. Quota sampling was used to achieve maximum ethnic similarity with the total sample. All participants (*n* = 9 and *n* = 10 from (different parts of) low- and high-deprivation areas, respectively) could speak English.

### 2.3. Measuring Outdoor Walking Level

For measuring participants’ outdoor walking levels, a GPS tracking unit (i-gotU GT-600) was used. All participants from low- and high-deprivation areas were trained and used the units (set on motion detector mode and 2-s recording interval) for a period of 3 to 8 days (*Mean* = 4.95, *SD* = 1.61), depending on their willingness and availability [37]. By using tracking units, detailed data on the location (*x*, *y*), date and time of participants’ outdoor walking activities were collected. By employing a GIS, each participant’s outdoor walking level was measured within a home-based neighborhood: a 2-km Euclidean buffer around each participant’s home [37]. All outdoor walking activities within this area were included in the measurement. For each participant, (average) outdoor walking level (minutes per day) was calculated in this way: (sum of durations of all walking activities)/(number of days that participant was loaned the GPS device).

### 2.4. Measuring Personal Characteristics

A questionnaire was used to collect data on six personal characteristics: age (65–74 years old or 75 years old and over); gender; marital status (single or in relationship); ethnicity (black and minority ethnic (BME) groups—i.e., Asian, Black, or mixed ethnic heritage—or white British [46]); educational attainment (sub-GCSE (General Certificates of Secondary Education or its equivalents) or GCSE and higher); and perceived health status over the last twelve months (poor or good). Missing data on each personal characteristic was less than 5%—except 11% missing data on educational attainment [37].

### 2.5. Measuring Neighborhood Built Environment Attributes

#### 2.5.1. GIS-Based Measurements 

A GIS (ArcGIS 10.4, ESRI, Redlands, CA, USA) and data presented in Table 2 were used to objectively measure neighborhood built environment attributes within each participant’s home-based neighborhood (the same area used for measuring outdoor walking levels).

We used Points of Interest (PoI) data to generate a land-use map, distinguishing residential and non-residential land uses. For this purpose, PoI was overlain with Topography Layer of OSMM and non-residential buildings were identified. Similar to a previous study [29], we considered one use for each building. After identifying non-residential buildings, we digitized plots relevant to non-residential buildings. By excluding non-residential plots and buildings within these plots, residential buildings were identified and residential plots were digitized. Boundaries of all residential and non-residential plots were cross-referenced with Google Earth images. To identify “public” green spaces, we cross-referenced “recreational lands” (generated from PoI) with data on open spaces provided by Birmingham City Council [50].

To measure neighborhood residential density, we used data on number of household space at LSOA level [51], because each home-based neighborhood contains and also intersects with several LSOAs. To calculate neighborhood residential density, we used the following equation (where RD = residential density for a home-based neighborhood, *i* = the LSOA, D*_i_* = residential density of the LSOA (total number of household space in the LSOA/total LSOA’s residential land-use area (hectare)), *k_i_* = the proportion of the LSOA’s residential land-use area (hectare) located within a home-based neighborhood against total LSOA’s residential land-use area (hectare), *n* = the number of LSOAs):

$$RD=\;\overset{n}{\underset{i=1}{\sum }}k_{i}D_{i}$$

To measure neighborhood land-use mix, we generated land-use entropy score which represents the degree of land-use diversity in a home-based neighborhood. For this purpose, like previous studies [26,32], we included residential land use and (five types of) non-residential land uses that may encourage daily outdoor walking (Table 3) and we applied an equation (see Supplementary Materials: Note S1) used in a UK study [49]. The entropy score ranged from 0 representing homogeneity (all land uses are of a single type) to 1 representing the most land-use diversity (the neighborhood is evenly distributed among all land-use categories) [16].

To measure land-use intensity, we considered 7 types of non-residential land uses that may encourage or discourage daily outdoor walking among older adults (Table 3). We measured area (hectare) of land covered by each type of uses within each participant’s home-based neighborhood (a 2-km Euclidean buffer). To compare intensity of different types of land uses in neighborhoods, we calculated the percentage of neighborhood land devoted to each type of use: (area (hectare) of each type of land use/total area (hectare) of the home-based neighborhood) × 100.

To measure street connectivity, we used ITN layer and UP Theme layer of OSMM (Table 2). These layers of OSMM topographically represent roads and urban paths as links and the junctions as nodes [49]. We used the method explained by Stockton, Duke-Williams, Stamatakis, Mindell, Brunner and Shelton [49]—we combined ITN and UP networks by using the Network Analyst extension in ArcGIS—and created a pedestrian route network dataset. Motorways and slip roads were excluded from this network dataset, since they are forbidden routes for pedestrians in the UK [65]. Junction density was used as an indicator for neighborhood street connectivity [16,66]. We counted number of junctions (points identified from the pedestrian route network dataset) that connecting three or more roads/paths within participants’ home-based neighborhoods [49] and we calculated neighborhood street connectivity in this way: the number of junctions in a home-based neighborhood/the area (hectare) of the home-based neighborhood [16].

For neighborhood retail density, the area (hectare) of retail buildings and plots were measured within participants’ home-based neighborhoods. The neighborhood retail density was calculated being area of retail buildings in a home-based neighborhood/total area of retail plots in a home-based neighborhood [16].

Data on each neighborhood built environment attribute was produced and was exported to a statistical software (SPSS 24, IBM, Armonk, NY, USA) for statistical analyses. Similar to previous studies [32,67], we did not combine neighborhood built environment attributes to create a single composite “walkability index”, in the hope to better distinguish the respective role of each neighborhood built environment attribute—and subsequently, spatial inequalities—in high- and low-deprivation areas.

#### 2.5.2. Walking Interview

Walking interviews are an ideal technique for collecting rich qualitative data on perceived neighborhood built environment [68,69]. We conducted individual open-question walking interviews with participants from low- and high-deprivation areas (Table 4). Participants were informed about the purpose of the research. The interviews were performed in English. A GPS unit and a digital recorder were used for recording data. Participants were asked to determine walking routes to take the interviewer around the neighborhood and to explain about advantages and disadvantages of their neighborhoods for walking. Through walking interviews, participants were enabled to express their assessments of their neighborhoods’ built environment and to provide information on how their neighborhoods support them to take outdoor walks. They talked about their neighborhoods’ facilities and explained: how these facilities encourage/discourage them to take outdoor walks; how they get to different destinations; and how they move from one place to another place in their neighborhoods. Participants also showed us examples of different issues that they were talking about. The interviews lasted 30 to 60 min, depending on participants’ willingness to walk.

### 2.6. Data Analysis

#### 2.6.1. Quantitative Analysis 

Descriptive statistics was used to analyze the participants’ personal characteristics. The spatial distributions of outdoor walking levels and (objectively measured) neighborhood built environment attributes were analyzed using GIS. For this purpose, Natural Breaks in data sets were used to classify data in three levels (e.g., low, medium, and high).

We used independent sample *t*-tests to compare the average outdoor walking levels, as well as (objectively measured) neighborhood built environment attributes, between low- and high-deprivation areas.

To study the relationships between neighborhood built environment attributes and outdoor walking levels, we applied a statistical approach used in previous studies [32,37]: we employed hierarchical linear regression analyses and we examined each neighborhood built environment attribute (i.e., residential density, land-use mix, intensity of different types of land uses, street connectivity, and retail density) individually. In each regression model, we tested the interaction between the neighborhood built environment attribute and area deprivation. When the interaction was significant, analyses were conducted for low- and high-deprivation areas separately.

We controlled each regression model for two personal characteristics (i.e., marital status and ethnicity), since only these two personal characteristics were significantly related to outdoor walking levels [37]. Comparing to single or BME groups, participants who were in a relationship or white British were more likely to walk outside home (correlations between these two personal characteristics and objectively measured neighborhood built environment attributes were tested and reported in Supplementary Materials: Table S1). In all regression models the missing data was excluded listwise and logarithmic transformation was applied on all variables (*x* + *1*) to reduce heteroscedasticity. All statistical analyses were conducted considering a *p*-value < 0.05 as significant. There was no significant difference between averaged GPS lending period (number of days) in low- and high-deprivation areas [37].

#### 2.6.2. Qualitative Analysis

We used qualitative analyses to examine participants’ perceptions of the same neighborhood built environment attributes (i.e., residential density, land-use mix and intensity, street connectivity, and retail density) in order to triangulate and corroborate [70] the quantitative results. Thus, we used a deductive approach for the qualitative study. First, we conducted open coding to ensure that the important aspects of the qualitative data were not missed [71]. Then, we followed a thematic analysis approach [72] and defined four main themes (i.e., residential density, land-use mix and intensity, street connectivity and retail density). Codes were categorized by linking them to the themes. To improve the reliability of analysis, we continued the process until data analysis reached saturation. We rechecked the consistency of coding by repeating the process [73]. All process was done by employing a Computer Aided Qualitative Data Analysis (CAQDAS) software (ATLAS.ti Scientific Software Development GmbH, Berlin, Germany).

## 3. Results

### 3.1. Sample Characteristics

Sample characteristics are reported in Table 5. This table shows that the majority of participants from high-deprivation areas was from BME groups and/or had low educational attainment (sub-GCSE), while most participants from low-deprivation areas were white British and/or had high educational attainment (GCSE and higher). Moreover, in both low-and high-deprivation areas over 90% of participants perceived good health status.

### 3.2. Disparities in Outdoor Walking Levels between Low- and High-Deprivation Areas

**Figure 2:**
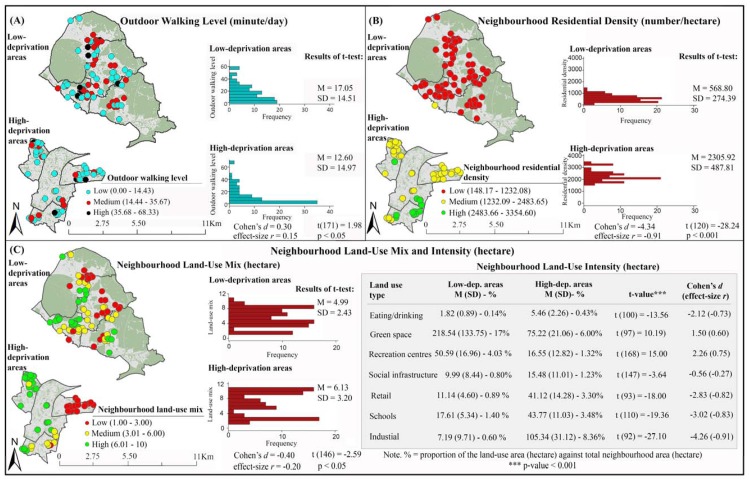

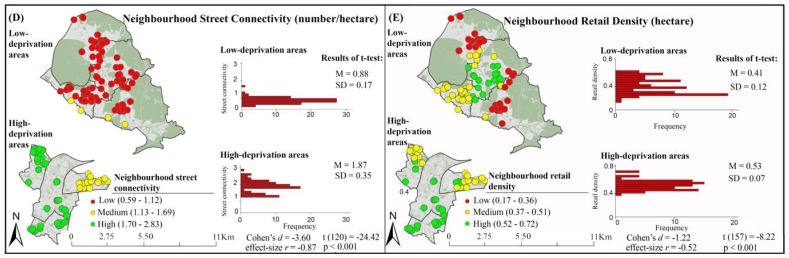
Disparities in outdoor walking levels and inequalities in objectively measured neighborhood built environment attributes (OS open data Boundary-line © Crown copyright/database right 2012 and OS MasterMap data © Crown Copyright/database right 2016. An Ordnance Survey/EDINA Digimap supplied service). Data on outdoor walking levels (box A) is from [37]. Each point shows location of a participant’s home. Frequency = number of participants; M = Mean; SD = Standard Deviation; *t* = *t*-value.

Outdoor walking levels in low- and high-deprivation areas are illustrated in Figure 2A. The minimum, maximum and average outdoor walking levels are 0.00, 68.33 and 14.99 min/day respectively. Compared to low-deprivation areas, high and medium walking levels are less prevalent in high-deprivation areas (Figure 2A). The results of *t*-test also indicate that (on average) participants from high-deprivation areas walk outside home significantly less than their peers from low-deprivation areas (Figure 2A).

### 3.3. Spatial Inequalities in Objectively Measured Neighborhood Built Environment Attributes 

Spatial inequalities in neighborhood residential density, land-use mix and intensity, street connectivity, and retail density are presented in Figure 2. High residential density (Figure 2B), high land-use mix (Figure 2C), high street connectivity (Figure 2D), and high retail density (Figure 2E) are more prevalent in high-deprivation areas than in low-deprivation areas. Moreover, intensities of specific types of land uses (i.e., eating/drinking, social infrastructure, retail, schools, and industries) are higher in high-deprivation areas than in low-deprivation areas (Figure 2C). Inverse trends were found for intensities of other types of land uses (i.e., green space and recreation centers). While in high-deprivation areas, a large percentage (8%) of neighborhood land is devoted to industries, in low-deprivation areas, a large percentage (17%) of neighborhood land is devoted to green space (Figure 2C). The results of *t*-test show that differences in neighborhood built environment attributes between low- and high-deprivation areas are significant (Figure 2).

### 3.4. Relationships between Neighborhood Built Environment Attributes and Outdoor Walking Levels

Neighborhood land-use intensity was related to outdoor walking levels. Intensities of two types of land uses (i.e., green space and recreation centers) were positively—and intensities of two types of land uses (i.e., schools and industries) were negatively—related to outdoor walking levels (Table 6). Therefore, participants living in neighborhoods where greater land area is dedicated to green space and recreation centers or where lesser land area is dedicated to schools and industries are more likely to take longer outdoor walks. Surprisingly, neighborhood street connectivity was negatively related to outdoor walking levels (Table 6). It means that participants living in neighborhoods with more number of junctions are less likely to walk outside home. Neighborhood residential density, land-use mix, and retail density were not related to outdoor walking levels.

The interactions between area deprivation and neighborhood land-use mix and intensity (i.e., intensity of social infrastructure) were significantly related to outdoor walking levels (Table 6). Table 7 shows that land-use mix is related to outdoor walking levels only in low-deprivation areas. However, intensity of social infrastructure was not related to outdoor walking levels in low- and high-deprivation areas. This finding indicates that relationships between intensity of social infrastructure and outdoor walking levels are similar in low- and high-deprivation areas.

### 3.5. Qualitative Results on Perceived Neighborhood Built Environment Attributes

Qualitative findings provide evidence on perceived neighborhood residential density, land-use mix and intensity, street connectivity, and retail density. They show that generally the participants living in high-deprivation areas perceive more built environment challenges, especially in terms of neighborhood land-use intensity, for outdoor walking. The following subsections explain the qualitative findings in detail.

#### 3.5.1. Residential Density

Participants from both low- and high-deprivation areas reported that they walk to their friends’ or relatives’ homes because their friends/relatives’ homes are located at close distances. Nevertheless, participants from high-deprivation areas discussed that there are many people and houses in these neighborhoods, which result in more social disorder, less beautiful scenery, and fewer local green spaces for outdoor walking: “You can see, this is a very congested area. (…) larger families are living in this area. When the summer comes, many children come in the street (…) there isn’t any youth activity in the area. They (children and youth) do not know where to go. They hang here and there, smash the windows and create problems in the area (…) For the elderly people, we don’t have really any place (…) We need more areas like parks” (a participant, high-deprivation areas).

Participants from low-deprivation areas, however, did not discuss challenges related to residential density. Interestingly, a participant from low-deprivation areas said that the residential area is suitable for taking a recreational walk with a dog: “I usually walk that way, where all the houses are, because I walk with the dog and it is not easy to take the dog to Mere Green, where the shops are, because I can’t leave him anywhere to go shopping.”

#### 3.5.2. Land-Use Mix and Intensity

Participants outlined three issues related to neighborhood land-use mix and intensity: (1) un/availability of destinations/places; (2) distance to destinations; and (3) attractiveness of destinations/places.

*(1) Un/availability of destinations/places:* All participants talked about walking to non-residential destinations (e.g., shop and mosques) in their neighborhoods. Nevertheless, they discussed about lack of some facilities (i.e., green spaces and recreation centers (e.g., gym) in high-deprivation areas; and libraries and shops in some parts of low-deprivation areas (e.g., Hill Hook)). The absence of social infrastructure—especially community centers with activities for older adults—was reported in high-deprivation areas and some parts of low-deprivation areas (e.g., Hill Hook and New Oscott). Participants explained that they may be encouraged to take more outdoor walks if these facilities (i.e., green spaces and gyms, or libraries and shops, or community centers with activities for older adults) are provided in their neighborhoods: “If there was a community center for people over 65, I’d go out maybe every day” (a participants, high-deprivation areas).

Presence of schools and industries discouraged outdoor walking. As participants explained, schools generate traffic and traffic hazards for walking: “There is a school (…) because of the school, the traffic is all over here (…) you’ve got a lot of children being brought to the school by car, not so many walking, (…) this area around here is very dangerous (for walking)” (a participant, low-deprivation areas).

Presence of industries provides unattractive scenery and makes neighborhoods boring for walking. Presence of many schools and industries was reported in high-deprivation areas. Participants from low-deprivation areas did not talk about industries in their neighborhoods.

*(2) Distance to destinations:* Participants from both low- and high-deprivation areas pointed to “close distance” to destinations (e.g., shops or places of worship) as an important issue for outdoor walking. Most participants from low- and high-deprivation areas were satisfied with the (perceived) distances to available facilities in their neighborhoods. A participant from a low-deprivation area said, “It (walking) is convenient to shops up the Mere Green and that encourages you to walk that distance”. Participants from low- and high-deprivation areas were also satisfied with hilly distances to available destinations and explained that they do not avoid hills if they have to face them.

*(3) Attractiveness of destinations/places:* Notwithstanding the presence of specific facilities (i.e., parks, shops, restaurants and cafés) at close distances, participants from high-deprivation areas perceived them as unattractive destinations for walking. They explained that available green spaces in their neighborhoods are small, dirty, unsafe and not interesting (except a couple of parks, such as Aston Park) and do not motivate them to walk. A participant said: “(To encourage me to walk) you can refurbish the park. (…) We’ve got a park (Ward End Park), but it could be improved. We don’t have a (high quality) park!” Some participants also discussed that some facilities (e.g., groceries and clothing shops) do not provide acceptable products and are not considered as walking destinations. A White British participant said: “shops are within easy walking distance of my home but they are not the shops I want, they are Asian!”. Some facilities (i.e., restaurants and cafés) were perceived unaffordable for some participants and did not encourage these participants to walk.

In contrast, in low-deprivation areas, the participants were satisfied with attractiveness of destinations for walking (e.g., shops, café and restaurants). They explained how a high quality of facilities such as green spaces (e.g., Sutton Park and New Hall Valley) encourages them to take outdoor walks. They said the large size and the history of green spaces, the beautiful nature, the variety of nice sceneries, the access to different gates, the lights and facilities (e.g., café and restaurant) and safety give them plenty of opportunities and incentives for walking for recreational purposes. Moreover, green spaces offer them beautiful shortcuts and some participants walk through the green spaces to get to their destinations. A participant said: “I mean the park is seven square miles and it has five lakes. It has at least six gates to get into it, from the various angles. (…) I use it (as a shortcut) if I go to travel. (…) you spend three hours for (recreational) walking (around the park) and then you can have a coffee and a lunch.”

#### 3.5.3. Street Connectivity

Participants from both low- and high-deprivation areas were satisfied with moving from one place to another place through different routes or shortcuts in their neighborhoods. They explained that presence of alternative routes and shortcuts facilitates their movement in their neighborhoods and encourages them to get to their destinations on foot. A participant from low-deprivation areas said: “We’ll do a short cut into the (New Hall) Valley (…) there are one or two cul-de-sacs (…) but in most places there is a cut-through somewhere, we’re going to do another one (shortcut)”. A participant from high-deprivation areas also said: “You can get from A to Z (in my neighborhood), you can go many ways. You can walk many ways!”

#### 3.5.4. Retail Density 

Participants explained that there are spaces devoted to cars (e.g., parking) next to some retail units. However, presence of these spaces was not perceived as a challenge for outdoor walking. Some participants from low- and high-deprivation areas benefit from presence of these spaces (i.e., parking), because they use a car to go shopping at close distances in order to avoid carrying heavy bags: “If I go to the small shops, I will walk. If I need a big shop, once a week in Sainsbury’s (supermarket), I will take a car, because I have to carry them” (a participant, low-deprivation areas). Presence of parking by retails facilitates shopping for these participants and encourages participants to use these facilities. Although these participants use a car to go shopping, they do it due to their reluctance to carry heavy shopping bags, not due to distances to the shops or traffic hazards.

### 3.6. Combining Quantitative and Qualitative Results

A combination of quantitative and qualitative results is presented in Table 8. Quantitative results indicate that intensities of specific land uses (i.e., green space and recreation centers) in neighborhoods are positively—and intensities of specific land uses (i.e., schools and industries) are negatively—related to outdoor walking levels. These results are supported by qualitative results showing that presence of (attractive) green space and recreation centers (e.g., gyms) encourages—and presence of schools and industries discourages—outdoor walking. Quantitative results also show spatial inequalities in land-use intensity (lower intensities of green space and recreation centers, and higher intensities of schools and industries) in high- versus low-deprivation areas. These findings are consistent with qualitative results showing (perceived) lack of green space and recreation centers—and presence of many schools and industries—in high-deprivation areas. Combining the quantitative and qualitative results indicates that inequalities in intensities of specific neighborhood land uses (i.e., green space, recreation centers, schools, and industries) in high- versus low-deprivation areas may influence the disparities in participants’ outdoor walking levels.

Neighborhood street connectivity was negatively related to outdoor walking levels. However, this result is not consistent with qualitative results showing that (perceived) short and diverse routes encourage outdoor walking in both low- and high-deprivation areas. Therefore, in this study, inequalities in neighborhood street connectivity (i.e., short and diverse routes) in high- versus low-deprivation areas do not influence the disparities in participants’ outdoor walking levels. We discuss about this issue later in this study. Neighborhood land-use mix was related to outdoor walking levels only in low-deprivation areas. Neighborhood residential density and retail density were not related to outdoor walking levels.

## 4. Discussion

This study examined inequalities in neighborhood walkability (i.e., residential density, land-use mix and intensity, street connectivity, and retail density) in high- versus low-deprivation areas and their possible influences on older adults’ outdoor walking levels in Birmingham, UK. Consistent with previous studies [6,8], it showed that participants from high-deprivation areas walk outside home less than their peers from low-deprivation areas. It demonstrated that inequalities in neighborhood land-use intensity (i.e., intensities of green space, recreation centers, schools and industries) in high- versus low-deprivation areas might influence disparities in participants’ outdoor walking levels between these areas. The following subsections discuss about findings of this study.

### 4.1. Neighborhood Walkability in Low- and High-Deprivation Areas

This study showed that many aspects of neighborhood walkability are more predominant in high-deprivation areas than in low-deprivation areas. Higher neighborhood residential density, land-use mix, street connectivity, and retail density were found in high-deprivation areas than in low-deprivation areas. These results are consistent with previous studies on adults showing higher land-use mix [32], street connectivity [25,32], and composite walkability index [21] in high- versus low-deprivation areas. Consistent with a previous study on adults [25], this study showed that only intensity of certain neighborhood land uses (i.e., green space and recreation centers) is lower in high-deprivation areas than in low-deprivation areas. Therefore, in high-deprivation areas, the structure of neighborhoods for outdoor walking is partially suitable for outdoor walking: for example, short and diverse routes exist in high-deprivation areas. King and Clarke [21] have addressed it as a strength of some high-deprivation areas. Considering findings of this study, policy makers/urban planners may need to modify land-use intensity (e.g., increasing green space intensity and decreasing industry intensity) in these areas. Identifying correlations between intensities of different types of land uses in neighborhood may help in modifying neighborhood land-use intensity.

### 4.2. Neighborhood Walkability and Outdoor Walking Levels 

Despite high levels of many aspects of neighborhood walkability in high-deprivation areas, participants living in these areas walked less than their peers living in low-deprivation areas. Consistent with previous studies on older adults [54,59], this study showed that intensities of specific land uses (i.e., green space and recreation centers) in neighborhoods are related to outdoor walking levels. However, neighborhood residential density, and retail density are not related to outdoor walking levels. Furthermore, intensities of schools and industries (that occupy a large percentage of neighborhood land in high-deprivation areas), and neighborhood street connectivity are negatively related to outdoor walking levels. These findings support previous studies [32,63] addressing industries as unsuitable land use for walking, Berke, Koepsell, Moudon, Hoskins and Larson [39]’s discussion on negative associations between schools and neighborhood walkability, and findings of a previous study [74] on negative associations between street connectivity and older adults’ walking. The relationships between neighborhood built environment attributes and outdoor walking levels were not moderated by area deprivation, except for neighborhood land-use mix, that is related to outdoor walking levels only in low-deprivation areas. A previous study on adults’ physical activity has reported similar results on composite walkability index in Ghent, Belgium [23].

Concerning the qualitative results, the insignificant or negative relationships between some neighborhood built environment attributes and outdoor walking levels may be influenced by other neighborhood built environment attributes. For example, relationships between neighborhood residential density, intensities of schools and industries, and outdoor walking levels may be influenced by social disorder, traffic conditions and aesthetics in neighborhoods (Table 8). Moreover, negative relationship between neighborhood street connectivity (i.e., number of junctions) and outdoor walking levels is probably influenced by other factors, such as traffic hazards at junctions [74] and higher level of traffic noise at junctions [75] that may be negatively related to older adults’ outdoor walking levels [37]. Furthermore, in line with other studies [8], it was qualitatively shown that intensities of some types of land uses (i.e., green spaces, eating/drinking and retail) may provide destinations, but these destinations are not attractive for walking (Table 8). Previous studies have also discussed that attractiveness of destinations is important for walking [28]. Moreover, other neighborhood built environment attributes (i.e., safety, pedestrian infrastructure such as traffic condition and pavement conditions, and aesthetics such as presence of trees and greenery) may influence outdoor walking [17]. Therefore, it is likely that other neighborhood built environment attributes (e.g., safety, pedestrian infrastructure and aesthetics)—and unattractiveness of destinations/places—weaken or negate positive impacts of macro built environment attributes (e.g., presence of friends’ homes at close distance and presence of short and diverse routes to destinations) on outdoor walking levels in high-deprivation areas. Additionally, a review study done by Trost, et al. [76] has found that in addition to neighborhood built environment attributes, social/cultural environment, such as social support, and some individual factors, such as lack of time and self-efficacy, may influence participation in physical activity.

Although this study focused on macro built environment attributes, it paves the way for future research: (1) to examine influences of other neighborhood built environment attributes (e.g., neighborhood safety and noise level) on older adults’ outdoor walking levels in low- and high-deprivation areas; (2) to investigate possible correlations between all neighborhood built environment attributes (e.g., neighborhood residential density, street connectivity, aesthetics and noise) influencing older adults’ outdoor walking; (3) to study relationships between attractiveness of non-residential destinations (e.g., green spaces) and older adults’ outdoor walking levels in low- and high-deprivation areas; and (4) to study the influences of social/cultural environment and individual factors (e.g., lack of time and self-efficacy) on older adults’ outdoor walking levels in low- and high-deprivation areas. Moreover, this study did not address outdoor walking for different purposes (e.g., transport and recreation) due to lack of data on purpose of outdoor walking. It is likely that neighborhood built environment attributes (e.g., street connectivity) differently associate with older adults’ outdoor walking levels for different purposes [77]. Future studies may improve knowledge on relationships between neighborhood built environment attributes and older adults’ outdoor walking levels for different purposes in low- and high-deprivation areas. Although this study addressed land-use intensity (measured by land area), it did not investigate number of each type of destinations. A greater (or lesser) area (hectare) of certain types of land uses does not necessarily mean a higher (or lower) number of those types of destinations. Future studies may examine influences of number of destinations (e.g., retail, green space, recreation centers, and facilities such as bus stops) on older adults’ outdoor walking levels in low- and high-deprivation areas.

### 4.3. Limitations

This study has some limitations. Data from different years (e.g., outdoor walking levels from 2012 and layers of OSMM from 2016) was used in this study. Moreover, this study considered one use of each building for generating a land-use map (it did not involve vertical development: tall multi-use buildings were treated in the same way as one-storey buildings). A coarse data on residential land use was used: identifying residential use by excluding non-residential uses did not involve residential use in multi-use buildings. This study assumed that there is a pavement in each road/street and generated the pedestrian network by combining ITN and UP networks (“the drivable road network” and “path network suitable for non-vehicular users”). It did not involve presence or absence of pavements in generating the pedestrian network due to lack of data. Cross-sectional nature of study prevented this study from making a causal inference. This study was done in one UK city with a convenience sample; therefore, participants may not be representative of all older residents, especially older residents with poor health status. Self-selection bias is a probability: people who enjoy walking may choose to live in neighborhoods that support walking. Difference between perceived neighborhood area and defined home-based neighborhood is also a probability. Nevertheless, this study provides an insight into the spatial inequalities in low- and high-deprivation areas which is applicable to more heterogeneous samples, other cities and future research.

## 5. Conclusions

This study extends the literature on neighborhood walkability, especially for older adults. It enriches the existing knowledge of influences of spatial inequalities in the built environment (in high-versus low-deprivation areas) on physical activity levels. It is one of the first examples of research on older adults’ outdoor walking combining a spatial inequality approach, GIS and GPS technology, and participants’ perceptions. It showed that spatial inequalities in one aspect of neighborhood walkability (i.e., neighborhood land-use intensity: intensities of green spaces, recreation centers, schools, and industries) in high- versus low-deprivation areas may influence the disparities in participants’ outdoor walking levels between these areas. This study may help policy makers and urban planners to determine how to improve neighborhood walkability for older adults, especially in high-deprivation areas. Land-use strategies aiming at modifying intensities of land uses in neighborhoods may help in supporting and encouraging outdoor walking in high-deprivation areas.

## Figures and Tables

**Table 1:** Definitions of neighborhood residential density, land-use mix and intensity, street connectivity, and retail density.

Neighborhood Built Environment Attributes	Definition
Residential density	Residential density refers to the number of dwellings in relation to the total amount of land devoted to residential use in a neighborhood [16,31]. Neighborhoods with higher residential density offer more residences (e.g., friends’ homes) as walking destinations at close distances [22].
Land-use mix	Land-use mix refers to the level of integration of diverse types of land uses in a neighborhood [22]. Studies on walkability usually address diverse types of land uses that may encourage walking [26,32]—such as residential, retail, and green space—and employ the land-use entropy to measure land-use mix [16]. Neighborhoods with more mixing of land uses offer diverse destinations (e.g., retails and green spaces) at close distances [22]. Land-use mix is used to identify the influences of a combination of diverse destinations on outdoor walking levels.
Land-use intensity	In this study, land-use intensity refers to the amount of land devoted to each type of use relative to the total land of the neighborhood. Land-use intensity is used to identify that presence or absence of what types of land use may encourage or discourage outdoor walking. High intensity of specific land uses may provide specific destinations or places for walking and may improve neighborhood walkability [33]. This aspect of neighborhood land use is not captured by using the land-use entropy [34].
Street connectivity	It refers to directness or easiness of moving between two points in a neighborhood [22]. Neighborhoods with high street connectivity have streets with many intersections and few cul-de-sacs. Such street networks provide short direct routes and make it easier to walk from an origin (e.g., home) to a destination (e.g., a shop) and also, offer a choice of taking different routes to the same destination [22]. Street connectivity is usually measured by junction density in a neighborhood [16].
Retail density	It refers to the amount of retail (i.e., all shops and stores) floor area in relation to the total amount of land devoted to retails in a neighborhood [31]. It is an indicator of compactness of retail area [32]. Neighborhoods with higher retail density provide less space devoted to cars (e.g., parking), more traffic safety, and shorter distances between retail building entrances, transit, and other activities [16].

**Table 2:** Data used for objective measures of neighborhood built environment attributes.

Neighborhood Built Environment Attributes	Data	Data Definition	Data Source
Residential density ^a^	Number of household spaces	Number of household spaces within each LSOA.	UK Census 2001 ^b^ Digimap/EDINA ^c^
	LSOAs boundary-line 2012	Polygons representing boundary of each LSOA.	
Land-use mix and intensity, and retail density	OS Points of Interest (PoI) 2016	Represents point locations of non-residential uses and delivers classification of these uses, such as retails, schools, etc. [47].	Digimap/EDINA ^c^
	Topography layer of OSMM 2016	The most detailed and accurate data on UK physical features, such as roads and buildings [48].	
Street connectivity	Integrated Transport Network (ITN) layer of OSMM 2016	Data on the drivable road network of the UK [49].	Digimap/EDINA ^c^
	Urban Path (UP) Theme layer of OSMM 2016	Data on the urban path network suitable for non-vehicular users, including all man-made footpaths, subways, steps, foot bridges and cycle path [49].	

Note. ^a^ To measure residential density, data on neighborhood land-use mix and intensity was also used for identifying residential land use. ^b^ This source was also used for the participant recruitment [37]. ^c^ Digimap/EDINA is the national data center for UK academics. OSMM = Ordnance Survey MasterMap.

**Table 3:** Description of non-residential land-use types.

Land-Use Types	Included Services and Facilities ^a^	Studies
May encourage daily outdoor walking:		
Eating/drinking	Cafes, snack bars and tea rooms; fast food and takeaway outlets; fast food delivery service; fish and chips; internet cafes; pubs, bars and inns; and restaurants.	[39,52]
Green spaces	Public parks and gardens; natural green spaces; and amenity green spaces.	[53,54,55,56]
Recreation centers	Athletic facilities, bowling facilities, golf courses, snooker and pool halls, squash courts, swimming pools, tennis facilities.	[57,58,59]
Social infrastructure	Halls and community centers; libraries; and places of worship; cinemas; nightclubs; social clubs; theatre and social halls.	[55,60,61]
Retail	All shops and stores selling cloth and accessories; food, drink and multi item retail; household, office, leisure and garden stuffs.	[39,40,57,62]
May discourage daily outdoor walking:		
Schools	Broad age range and secondary state schools; first, primary and infant schools; further education establishments; higher education establishment; independent and preparatory schools; pupil referral units; special schools and colleges; and unspecified and other schools.	[39]
Industries	All services and facilities related to manufacturing and productions (i.e., consumer products, executive industries, farming, foodstuffs, industrial features, industrial products).	[32,63]

Note. ^a^ [64], Services and facilities that may not relate to daily older adults’ outdoor walking (e.g., retail: motoring) were not included in land-use mix and intensity measurements.

**Table 4:** Detailed information about sub-sample (participants for walking interviews).

Participants’ Characteristics	Sub-Sample		
	Low-Deprivation Areas	High-Deprivation Areas	Total
Number of participants	9	10	19
Age (*n*):			
75 years old and over	5	5	10
65–74 years old	4	5	9
Gender (*n*):			
Men	2	4	6
Women	7	6	13
Marital status (*n*):			
In relationship	6	5	11
Single	3	5	8
Ethnicity (*n*):			
White British	8	5	13
BME groups	1	5	6
Educational attainment (*n*):			
GCSE and higher	9	2	11
Sub-GCSE	0	8	8
Health status (*n*):			
Good	9	9	18
Poor	0	1	1

Note. *n* = number. Data from [37].

**Table 5:** Sample characteristics in low- and high-deprivation areas and in total.

Participants’ Characteristics	Total Sample		
	Low	High	Total
Number of participants	93	80	173
Average age of participants (*M* (*SD*))	74.8 (5.82)	73.5 (5.95)	74.2 (5.90)
Age (%):			
75 years old and over	53	43	48
65–74 years old	47	57	52
Gender (%):			
Men	30	59	43
Women	70	41	57
Marital status (%):			
In relationship	53	53	53
Single	47	47	47
Ethnicity (%):			
White British	97	41	71
BME groups	3	59	29
Educational attainment (%):			
GCSE and higher	80	24	54
Sub-GCSE	10	64	35
Health status (%):			
Good	93	92	92
Poor	6	8	7

Note. Low = sample from low-deprivation areas; High = sample from high-deprivation areas; Total = sample from both low- and high-deprivation areas; *M* = Mean; *SD* = Standard Deviation. Data from [37].

**Table 6:** Results of hierarchical analyses: relationships between neighborhood built environment attributes and outdoor walking levels.

Neighborhood Built Environment Attribute	Outdoor Walking Levels	Interactions ^a^
	*B (SE)*	*B (SE)*
Residential density	−0.19 (0.12)	−0.09 (0.08)
Land-use-mix	0.01 (0.17)	−0.44 (0.17) *
Land-use intensity:		
Eating/drinking	−0.10 (0.12)	−0.08 (0.04)
Green space	0.28 (0.13) *	−0.03 (0.03)
Recreation centers	0.36 (0.13) **	0.01 (0.04)
Social infrastructure	−0.07 (0.12)	−0.29 (0.13) *
Retail	−015 (0.12)	−0.07 (0.04)
Schools	−0.44 (0.18) *	0.00 (0.04)
Industries	−0.13 (0.06) *	−0.01 (0.05)
Street connectivity	−0.45 (0.17) *	−0.17 (0.74)
Retail density	−0.58 (1.13)	−1.60 (0.87)

Note. Each neighborhood built environment attribute was examined individually. This table presents the results after controlling for personal characteristics (i.e., marital status and ethnicity). ^a^ Relationship between outdoor walking levels and interaction between area deprivation and the neighborhood built environment attribute. *B* = Unstandardized Coefficient; *SE* = Standard Error. The values in **bold** type are significant. * *p* < 0.05, ** *p* < 0.01.

**Table 7:** Results of hierarchical analyses: relationships between neighborhood land-use mix and intensity, and outdoor walking levels in low- and high-deprivation areas.

Neighborhood Land-Use Mix and Intensity	Outdoor Walking Levels	
	Low-Deprivation Areas	High-Deprivation Areas
	*B (SE)*	*B (SE)*
Land-use mix	0.47 (0.22) *	−0.30 (0.25)
Intensity of social infrastructure	0.11 (0.14)	−0.27 (0.25)

Note. This table presents the results after controlling for personal characteristics (i.e., marital status and ethnicity). *B* = Unstandardized Coefficient; *SE* = Standard Error. The value in **bold** type is significant. * *p* < 0.05.

**Table 8:** Combination of quantitative and qualitative results.

Neighborhood Built Environment Attribute	Quantitative Results		Qualitative Results
	Spatial Inequalities	Related to Walking ^a^ Levels	Perceived Influences of Neighborhood Built Environment Attributes on Outdoor Walking Levels
Residential density	High > Low	No	High: encouraged walking by providing close destination. Discouraged walking due to generating social disorder, less beautiful scenery and fewer open spaces. Low: encouraged walking by providing close destinations and offering a suitable area for recreational walks.
Land-use mix	High > Low	No ^b^	High and Low: close distance to diverse destinations/place was important and encouraged walking.
Land-use intensity Eating/drinking	High > Low	No	High: were perceived as unattractive destinations by some participants and did not support walking among them. Low: were perceived as attractive destinations and encouraged walking.
Green space	High < Low	Yes +	High: lack of green spaces as attractive destinations/places for walking discouraged walking. Low: presence of green spaces as attractive destinations/places for walking encouraged walking.
Recreation centers	High < Low	Yes +	High: lack of recreation centers in neighborhood did not support walking. Low: presence of recreation centers in neighborhood encouraged walking.
Social infrastructure	High > Low	No	High: lack of these destinations (i.e., community centers with social activities for older adults) did not support walking. Low: lack of these destinations (i.e., libraries and community centers with social activities for older adults) did not support walking.
Retail	High > Low	No	High: were perceived as unattractive destinations by some participants and did not support walking among them. Low: lack of these destinations (i.e., shops) in some areas discouraged walking.
Schools	High > Low	Yes −	High: presence of many schools discouraged walking due to generating traffic dangers. Low: presence of schools discouraged walking due to generating traffic dangers.
Industries	High > Low	Yes −	High: presence of many industries discouraged walking by offering unattractive scenery in the neighborhood. Low: presence of industries was not discussed by participants.
Street connectivity	High > Low	Yes −	High and Low: perceived short and alternative routes encouraged walking.
Retail density	High > Low	No	High and Low: presence of spaces devoted to cars (e.g., parking) was not perceived as a challenge for walking.

Note. ^a^ Outdoor walking; ^b^ Neighborhood land-use mix was related to outdoor walking levels only in low-deprivation areas. Low = low-deprivation areas; High = high-deprivation areas; + a positive relationship; − a negative relationship.

## Supplementary Materials

The following are available online at www.mdpi.com/1660-4601/14/7/740/s1, Note S1. Equation used for measuring land-use entropy score, Table S1: Correlations between personal characteristics and objectively measured neighborhood built environment attributes.
