# Supplementary material for: Do Inequalities in Neighborhood Walkability Drive Disparities in Older Adults’ Outdoor Walking?
Source: Int J Environ Res Public Health. 2017 Jul 7;14(7):740. doi: 10.3390/ijerph14070740 (PMC5551178; doi:10.3390/ijerph14070740)
Supplement: Supplementary file 1 [file ijerph-14-00740-s001.pdf]

International Journal of
Environmental Research and Public Health

MDPI

Article

Do Inequalities in Neighbourhood Walkability Drive Disparities in Older Adults' Outdoor Walking?

Razieh Zandieh, Johannes Flacke, Javier Martinez, Phil Jones and Martin van Maarseveen

Note S1. Equation used for measuring land-use entropy score.

The following equation was used (where LMX = land-use mix score for a home-based neighbourhood, i = the land-use, p_i = the proportion of the land area devoted to a specific land-use against the sum of the area of the land-uses of interest, n = the number of land-use categories):

$$\text{LMX} = \left[-1\sum_{i=1}^{n} p_i^{*}\ln(p_i)\right] \Big/ \ln(n)$$

Table S1: Correlations between personal characteristics and objectively measured neighbourhood built environment attributes.

Personal Characteristic	Residential Density	Land-Use Mix	Land-Use Intensity							Street Connectivity	Retail Density
			Eating/Drinking	Green Space	Sport Complex	Social Infrastructure	Retail	Schools	Industries		
Marital status	0.04	−0.12	0.04	0.01	0.07	0.08	−0.01	−0.02	−0.03	0.04	−0.04
	0.570	*0.116*	*0.570*	*0.860*	*0.360*	*0.304*	*0.872*	*0.800*	*0.723*	*0.612*	*0.594*
Ethnicity	−0.51	−0.05	−0.42	0.33	0.40	−0.19	−0.41	−0.46	−0.52	−0.25	−0.51
	0.000	*0.480*	*0.000*	*0.000*	*0.000*	*0.011*	*0.000*	*0.000*	*0.000*	*0.001*	*0.000*

Note. This table shows Pearson correlation values; p-values are in *italic*.
